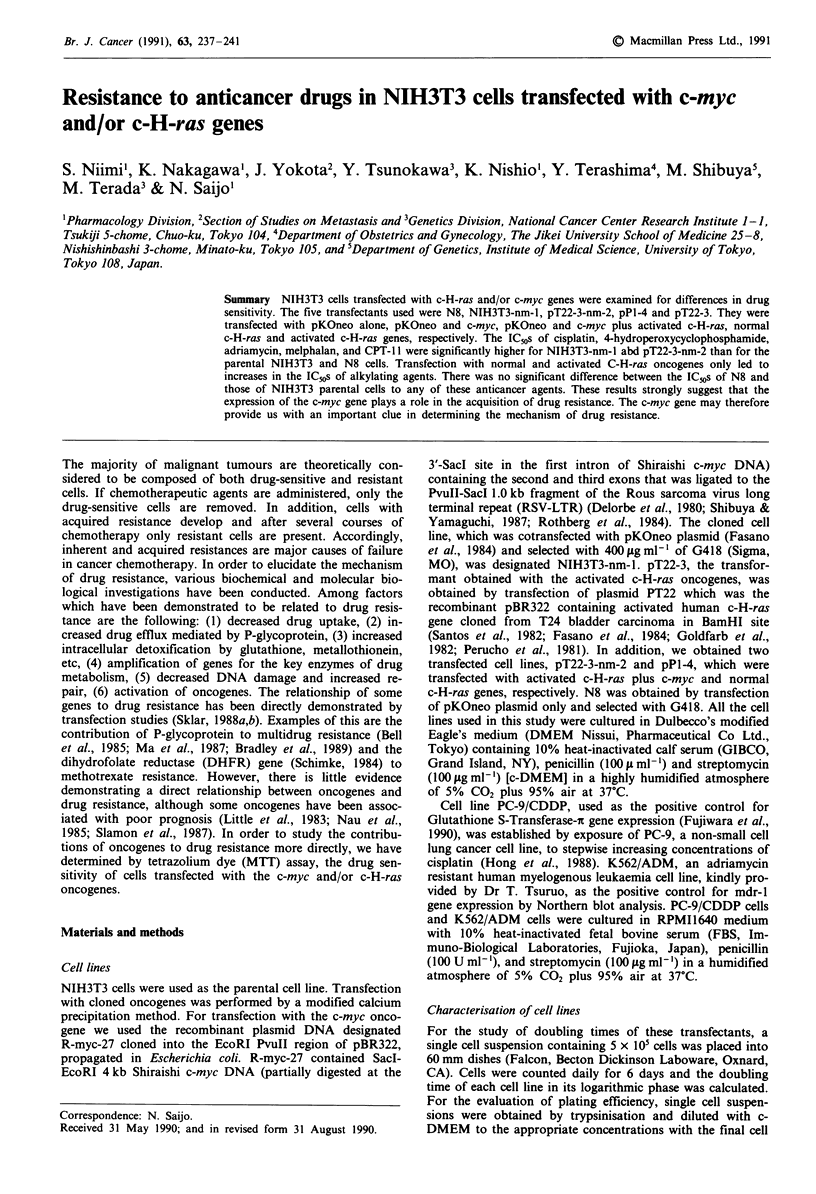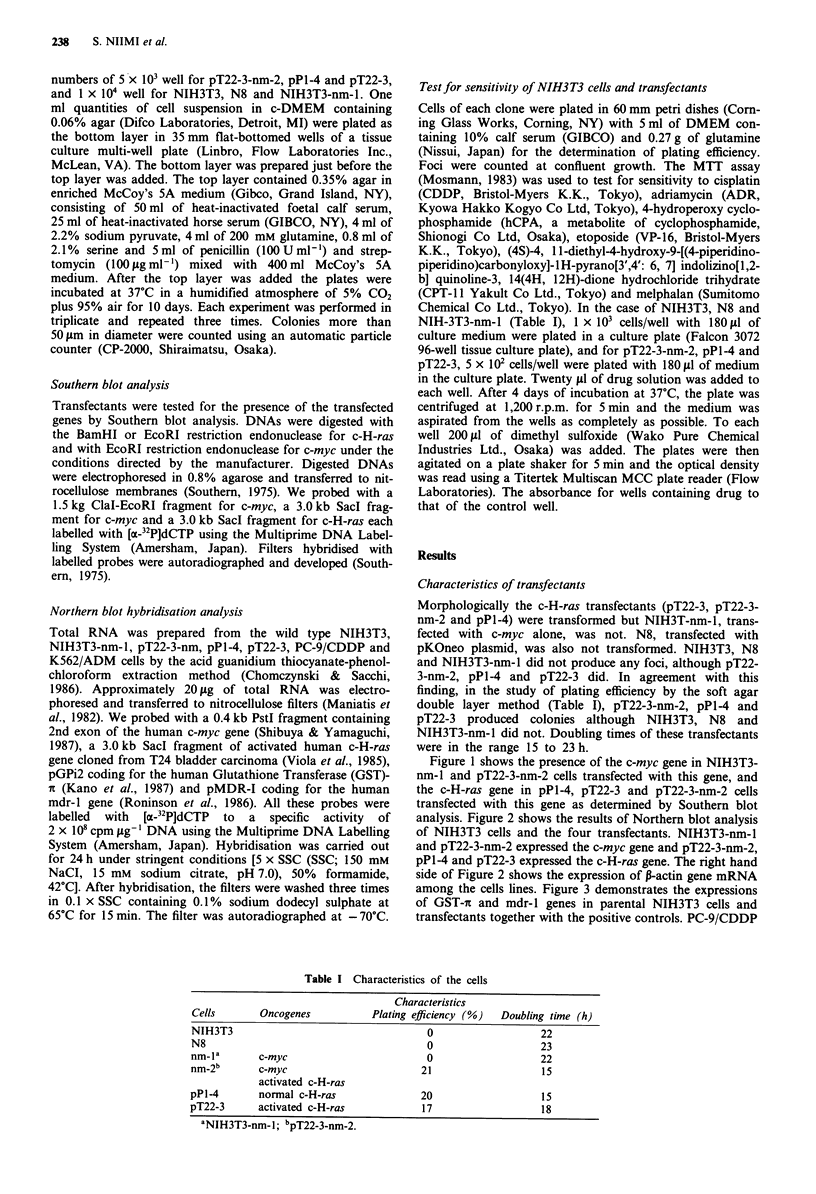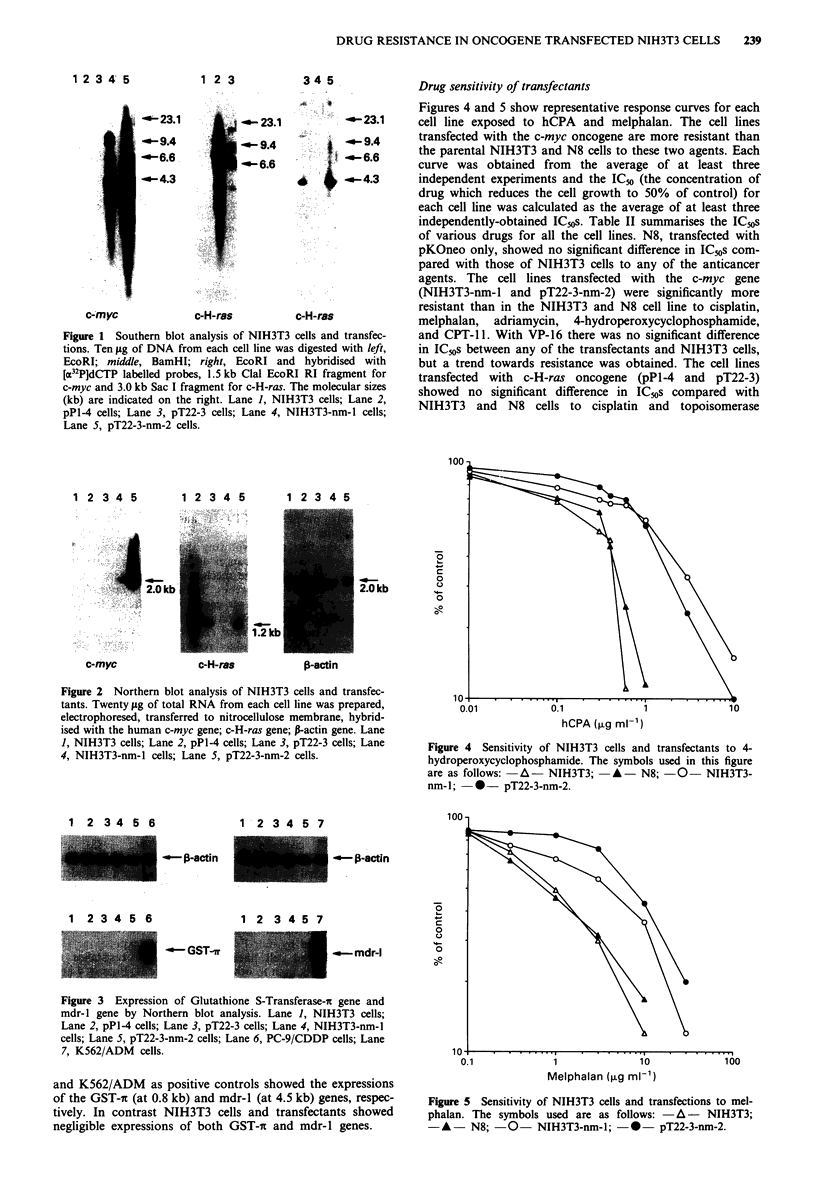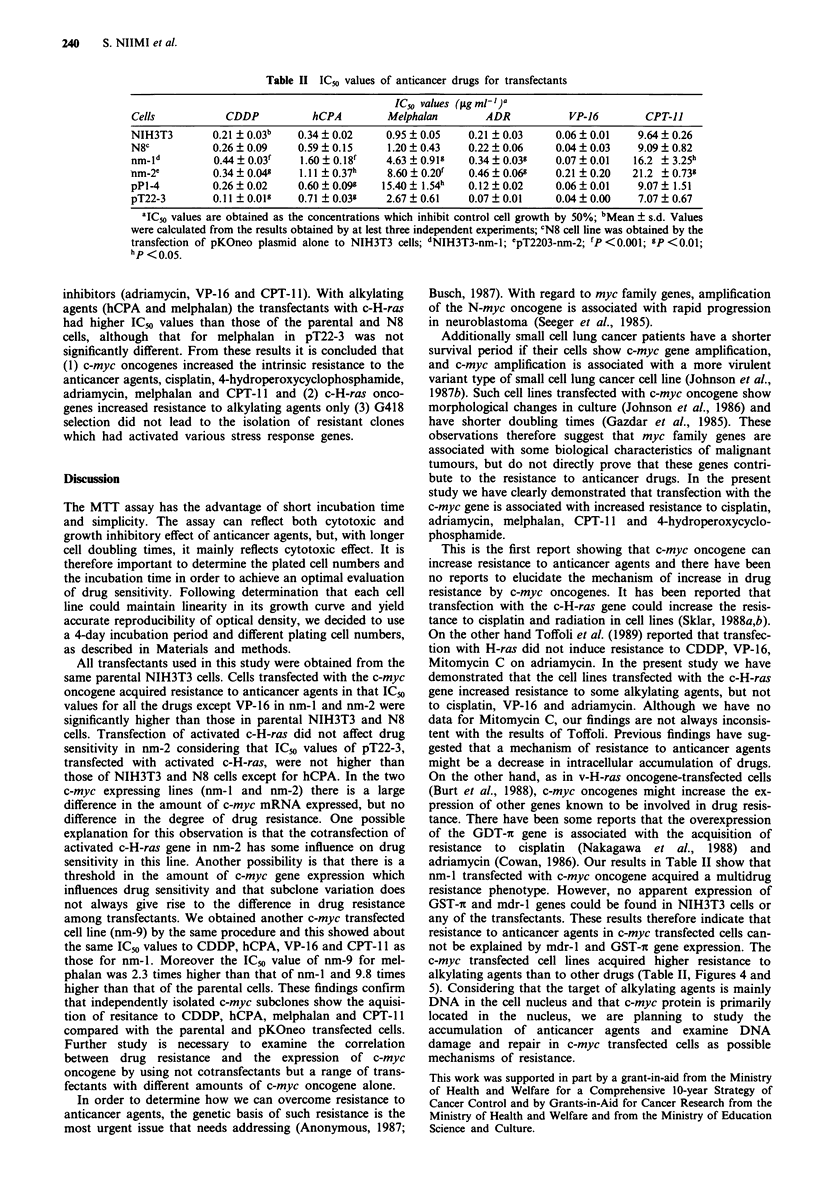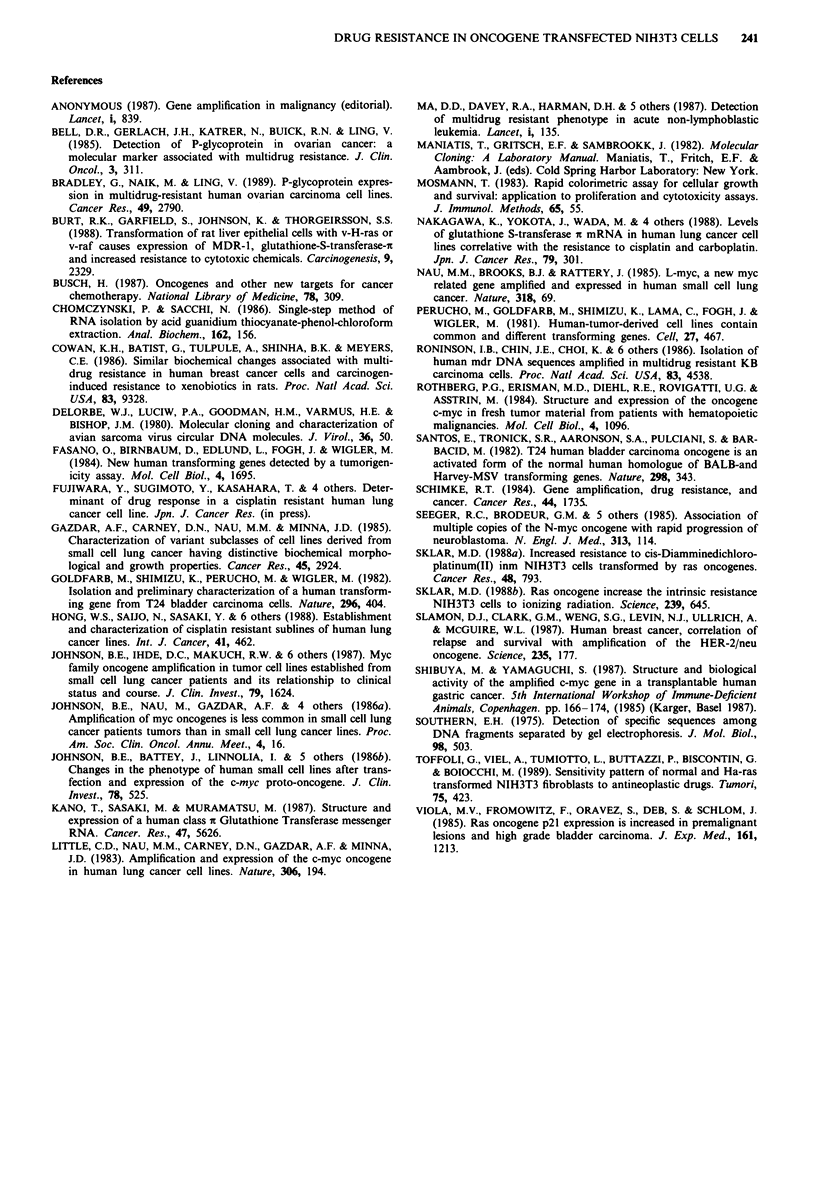# Resistance to anticancer drugs in NIH3T3 cells transfected with c-myc and/or c-H-ras genes.

**DOI:** 10.1038/bjc.1991.56

**Published:** 1991-02

**Authors:** S. Niimi, K. Nakagawa, J. Yokota, Y. Tsunokawa, K. Nishio, Y. Terashima, M. Shibuya, M. Terada, N. Saijo

**Affiliations:** Pharmacology Division, National Cancer Center Research Institute, Tokyo, Japan.

## Abstract

**Images:**


Br. J. Cancer (1991), 63, 237-241 © Macmillan Press Ltd., 1991

### Resistance to anticancer drugs in NIH3T3 cells transfected with c-*myc* and/or c-H-*ras* genes

S. Niimi[1], K. Nakagawa[1], J. Yokota[2], Y. Tsunokawa[3], K. Nishio[1], Y. Terashima[4], M. Shibuya[5], M. Terada[3] & N. Saijo[1]

[1]*Pharmacology Division*, [2]*Section of Studies on Metastasis and* [3]*Genetics Division, National Cancer Center Research Institute 1-1, Tsukiji 5-chome, Chuo-ku, Tokyo 104*, [4]*Department of Obstetrics and Gynecology, The Jikei University School of Medicine 25-8, Nishishinbashi 3-chome, Minato-ku, Tokyo 105, and* [5]*Department of Genetics, Institute of Medical Science, University of Tokyo, Tokyo 108, Japan.*

**Summary** NIH3T3 cells transfected with c-H-*ras* and/or c-*myc* genes were examined for differences in drug sensitivity. The five transfectants used were N8, NIH3T3-nm-1, pT22-3-nm-2, pP1-4 and pT22-3. They were transfected with pKOneo alone, pKOneo and c-*myc*, pKOneo and c-*myc* plus activated c-H-*ras*, normal c-H-*ras* and activated c-H-*ras* genes, respectively. The $IC_{50}$s of cisplatin, 4-hydroperoxycyclophosphamide, adriamycin, melphalan, and CPT-11 were significantly higher for NIH3T3-nm-1 abd pT22-3-nm-2 than for the parental NIH3T3 and N8 cells. Transfection with normal and activated C-H-*ras* oncogenes only led to increases in the $IC_{50}$s of alkylating agents. There was no significant difference between the $IC_{50}$s of N8 and those of NIH3T3 parental cells to any of these anticancer agents. These results strongly suggest that the expression of the c-*myc* gene plays a role in the acquisition of drug resistance. The c-*myc* gene may therefore provide us with an important clue in determining the mechanism of drug resistance.

The majority of malignant tumours are theoretically considered to be composed of both drug-sensitive and resistant cells. If chemotherapeutic agents are administered, only the drug-sensitive cells are removed. In addition, cells with acquired resistance develop and after several courses of chemotherapy only resistant cells are present. Accordingly, inherent and acquired resistances are major causes of failure in cancer chemotherapy. In order to elucidate the mechanism of drug resistance, various biochemical and molecular biological investigations have been conducted. Among factors which have been demonstrated to be related to drug resistance are the following: (1) decreased drug uptake, (2) increased drug efflux mediated by P-glycoprotein, (3) increased intracellular detoxification by glutathione, metallothionein, etc, (4) amplification of genes for the key enzymes of drug metabolism, (5) decreased DNA damage and increased repair, (6) activation of oncogenes. The relationship of some genes to drug resistance has been directly demonstrated by transfection studies (Sklar, 1988*a*,*b*). Examples of this are the contribution of P-glycoprotein to multidrug resistance (Bell *et al*., 1985; Ma *et al*., 1987; Bradley *et al*., 1989) and the dihydrofolate reductase (DHFR) gene (Schimke, 1984) to methotrexate resistance. However, there is little evidence demonstrating a direct relationship between oncogenes and drug resistance, although some oncogenes have been associated with poor prognosis (Little *et al*., 1983; Nau *et al*., 1985; Slamon *et al*., 1987). In order to study the contributions of oncogenes to drug resistance more directly, we have determined by tetrazolium dye (MTT) assay, the drug sensitivity of cells transfected with the c-*myc* and/or c-H-*ras* oncogenes.

### Materials and methods

### *Cell lines*

NIH3T3 cells were used as the parental cell line. Transfection with cloned oncogenes was performed by a modified calcium precipitation method. For transfection with the c-*myc* oncogene we used the recombinant plasmid DNA designated R-myc-27 cloned into the EcoRI PvuII region of pBR322, propagated in *Escherichia coli*. R-myc-27 contained SacI-EcoRI 4 kb Shiraishi c-*myc* DNA (partially digested at the 3'-SacI site in the first intron of Shiraishi c-*myc* DNA) containing the second and third exons that was ligated to the PvuII-SacI 1.0 kb fragment of the Rous sarcoma virus long terminal repeat (RSV-LTR) (Delorbe *et al*., 1980; Shibuya & Yamaguchi, 1987; Rothberg *et al*., 1984). The cloned cell line, which was cotransfected with pKOneo plasmid (Fasano *et al*., 1984) and selected with 400 $\mu g\ ml^{-1}$ of G418 (Sigma, MO), was designated NIH3T3-nm-1. pT22-3, the transformant obtained with the activated c-H-*ras* oncogenes, was obtained by transfection of plasmid PT22 which was the recombinant pBR322 containing activated human c-H-*ras* gene cloned from T24 bladder carcinoma in BamHI site (Santos *et al*., 1982; Fasano *et al*., 1984; Goldfarb *et al*., 1982; Perucho *et al*., 1981). In addition, we obtained two transfected cell lines, pT22-3-nm-2 and pP1-4, which were transfected with activated c-H-*ras* plus c-*myc* and normal c-H-*ras* genes, respectively. N8 was obtained by transfection of pKOneo plasmid only and selected with G418. All the cell lines used in this study were cultured in Dulbecco's modified Eagle's medium (DMEM Nissui, Pharmaceutical Co Ltd., Tokyo) containing 10% heat-inactivated calf serum (GIBCO, Grand Island, NY), penicillin (100 $\mu\ ml^{-1}$) and streptomycin (100 $\mu g\ ml^{-1}$) [c-DMEM] in a highly humidified atmosphere of 5% $CO_2$ plus 95% air at 37°C.

Cell line PC-9/CDDP, used as the positive control for Glutathione S-Transferase-π gene expression (Fujiwara *et al*., 1990), was established by exposure of PC-9, a non-small cell lung cancer cell line, to stepwise increasing concentrations of cisplatin (Hong *et al*., 1988). K562/ADM, an adriamycin resistant human myelogenous leukaemia cell line, kindly provided by Dr T. Tsuruo, as the positive control for mdr-1 gene expression by Northern blot analysis. PC-9/CDDP cells and K562/ADM cells were cultured in RPMI1640 medium with 10% heat-inactivated fetal bovine serum (FBS, Immuno-Biological Laboratories, Fujioka, Japan), penicillin (100 U $ml^{-1}$), and streptomycin (100 $\mu g\ ml^{-1}$) in a humidified atmosphere of 5% $CO_2$ plus 95% air at 37°C.

### *Characterisation of cell lines*

For the study of doubling times of these transfectants, a single cell suspension containing $5 \times 10^5$ cells was placed into 60 mm dishes (Falcon, Becton Dickinson Laboware, Oxnard, CA). Cells were counted daily for 6 days and the doubling time of each cell line in its logarithmic phase was calculated. For the evaluation of plating efficiency, single cell suspensions were obtained by trypsinisation and diluted with c-DMEM to the appropriate concentrations with the final cell

Correspondence: N. Saijo.
Received 31 May 1990; and in revised form 31 August 1990.

numbers of $5 \times 10^3$ well for pT22-3-nm-2, pP1-4 and pT22-3, and $1 \times 10^4$ well for NIH3T3, N8 and NIH3T3-nm-1. One ml quantities of cell suspension in c-DMEM containing 0.06% agar (Difco Laboratories, Detroit, MI) were plated as the bottom layer in 35 mm flat-bottomed wells of a tissue culture multi-well plate (Linbro, Flow Laboratories Inc., McLean, VA). The bottom layer was prepared just before the top layer was added. The top layer contained 0.35% agar in enriched McCoy's 5A medium (Gibco, Grand Island, NY), consisting of 50 ml of heat-inactivated foetal calf serum, 25 ml of heat-inactivated horse serum (GIBCO, NY), 4 ml of 2.2% sodium pyruvate, 4 ml of 200 mM glutamine, 0.8 ml of 2.1% serine and 5 ml of penicillin ($100\ U\ ml^{-1}$) and streptomycin ($100\ \mu g\ ml^{-1}$) mixed with 400 ml McCoy's 5A medium. After the top layer was added the plates were incubated at 37°C in a humidified atmosphere of 5% $CO_2$ plus 95% air for 10 days. Each experiment was performed in triplicate and repeated three times. Colonies more than 50 µm in diameter were counted using an automatic particle counter (CP-2000, Shiraimatsu, Osaka).

*Southern blot analysis*

Transfectants were tested for the presence of the transfected genes by Southern blot analysis. DNAs were digested with the BamHI or EcoRI restriction endonuclease for c-H-*ras* and with EcoRI restriction endonuclease for c-*myc* under the conditions directed by the manufacturer. Digested DNAs were electrophoresed in 0.8% agarose and transferred to nitrocellulose membranes (Southern, 1975). We probed with a 1.5 kg ClaI-EcoRI fragment for c-*myc*, a 3.0 kb SacI fragment for c-*myc* and a 3.0 kb SacI fragment for c-H-*ras* each labelled with [$\alpha$-$^{32}$P]dCTP using the Multiprime DNA Labelling System (Amersham, Japan). Filters hybridised with labelled probes were autoradiographed and developed (Southern, 1975).

*Northern blot hybridisation analysis*

Total RNA was prepared from the wild type NIH3T3, NIH3T3-nm-1, pT22-3-nm, pP1-4, pT22-3, PC-9/CDDP and K562/ADM cells by the acid guanidium thiocyanate-phenol-chloroform extraction method (Chomczynski & Sacchi, 1986). Approximately 20 µg of total RNA was electrophoresed and transferred to nitrocellulose filters (Maniatis *et al.*, 1982). We probed with a 0.4 kb PstI fragment containing 2nd exon of the human c-*myc* gene (Shibuya & Yamaguchi, 1987), a 3.0 kb SacI fragment of activated human c-H-*ras* gene cloned from T24 bladder carcinoma (Viola *et al.*, 1985), pGPi2 coding for the human Glutathione Transferase (GST)-$\pi$ (Kano *et al.*, 1987) and pMDR-I coding for the human mdr-1 gene (Roninson *et al.*, 1986). All these probes were labelled with [$\alpha$-$^{32}$P]dCTP to a specific activity of $2 \times 10^8$ cpm $\mu g^{-1}$ DNA using the Multiprime DNA Labelling System (Amersham, Japan). Hybridisation was carried out for 24 h under stringent conditions [5 × SSC (SSC; 150 mM NaCI, 15 mM sodium citrate, pH 7.0), 50% formamide, 42°C]. After hybridisation, the filters were washed three times in 0.1 × SSC containing 0.1% sodium dodecyl sulphate at 65°C for 15 min. The filter was autoradiographed at −70°C.

*Test for sensitivity of NIH3T3 cells and transfectants*

Cells of each clone were plated in 60 mm petri dishes (Corning Glass Works, Corning, NY) with 5 ml of DMEM containing 10% calf serum (GIBCO) and 0.27 g of glutamine (Nissui, Japan) for the determination of plating efficiency. Foci were counted at confluent growth. The MTT assay (Mosmann, 1983) was used to test for sensitivity to cisplatin (CDDP, Bristol-Myers K.K., Tokyo), adriamycin (ADR, Kyowa Hakko Kogyo Co Ltd, Tokyo), 4-hydroperoxy cyclophosphamide (hCPA, a metabolite of cyclophosphamide, Shionogi Co Ltd, Osaka), etoposide (VP-16, Bristol-Myers K.K., Tokyo), (4S)-4, 11-diethyl-4-hydroxy-9-[(4-piperidino-piperidino)carbonyloxy]-1H-pyrano[3',4': 6, 7] indolizino[1,2-b] quinoline-3, 14(4H, 12H)-dione hydrochloride trihydrate (CPT-11 Yakult Co Ltd., Tokyo) and melphalan (Sumitomo Chemical Co Ltd., Tokyo). In the case of NIH3T3, N8 and NIH-3T3-nm-1 (Table I), $1 \times 10^3$ cells/well with 180 µl of culture medium were plated in a culture plate (Falcon 3072 96-well tissue culture plate), and for pT22-3-nm-2, pP1-4 and pT22-3, $5 \times 10^2$ cells/well were plated with 180 µl of medium in the culture plate. Twenty µl of drug solution was added to each well. After 4 days of incubation at 37°C, the plate was centrifuged at 1,200 r.p.m. for 5 min and the medium was aspirated from the wells as completely as possible. To each well 200 µl of dimethyl sulfoxide (Wako Pure Chemical Industries Ltd., Osaka) was added. The plates were then agitated on a plate shaker for 5 min and the optical density was read using a Titertek Multiscan MCC plate reader (Flow Laboratories). The absorbance for wells containing drug to that of the control well.

### Results

*Characteristics of transfectants*

Morphologically the c-H-*ras* transfectants (pT22-3, pT22-3-nm-2 and pP1-4) were transformed but NIH3T-nm-1, transfected with c-*myc* alone, was not. N8, transfected with pKOneo plasmid, was also not transformed. NIH3T3, N8 and NIH3T3-nm-1 did not produce any foci, although pT22-3-nm-2, pP1-4 and pT22-3 did. In agreement with this finding, in the study of plating efficiency by the soft agar double layer method (Table I), pT22-3-nm-2, pP1-4 and pT22-3 produced colonies although NIH3T3, N8 and NIH3T3-nm-1 did not. Doubling times of these transfectants were in the range 15 to 23 h.

Figure 1 shows the presence of the c-*myc* gene in NIH3T3-nm-1 and pT22-3-nm-2 cells transfected with this gene, and the c-H-*ras* gene in pP1-4, pT22-3 and pT22-3-nm-2 cells transfected with this gene as determined by Southern blot analysis. Figure 2 shows the results of Northern blot analysis of NIH3T3 cells and the four transfectants. NIH3T3-nm-1 and pT22-3-nm-2 expressed the c-*myc* gene and pT22-3-nm-2, pP1-4 and pT22-3 expressed the c-H-*ras* gene. The right hand side of Figure 2 shows the expression of $\beta$-actin gene mRNA among the cells lines. Figure 3 demonstrates the expressions of GST-$\pi$ and mdr-1 genes in parental NIH3T3 cells and transfectants together with the positive controls. PC-9/CDDP

**Table I** Characteristics of the cells

| Cells | Oncogenes | Characteristics<br>Plating efficiency (%) | Doubling time (h) |
|---|---|---|---|
| NIH3T3 | | 0 | 22 |
| N8 | | 0 | 23 |
| nm-1[a] | c-*myc* | 0 | 22 |
| nm-2[b] | c-*myc*<br>activated c-H-*ras* | 21 | 15 |
| pP1-4 | normal c-H-*ras* | 20 | 15 |
| pT22-3 | activated c-H-*ras* | 17 | 18 |

[a]NIH3T3-nm-1; [b]pT22-3-nm-2.

**Figure 1** Southern blot analysis of NIH3T3 cells and transfections. Ten μg of DNA from each cell line was digested with *left*, EcoRI; *middle*, BamHI; *right*, EcoRI and hybridised with [$\alpha^{32}$P]dCTP labelled probes, 1.5 kb ClaI EcoRI RI fragment for c-*myc* and 3.0 kb Sac I fragment for c-H-*ras*. The molecular sizes (kb) are indicated on the right. Lane *1*, NIH3T3 cells; Lane *2*, pP1-4 cells; Lane *3*, pT22-3 cells; Lane *4*, NIH3T3-nm-1 cells; Lane *5*, pT22-3-nm-2 cells.

**Figure 2** Northern blot analysis of NIH3T3 cells and transfectants. Twenty μg of total RNA from each cell line was prepared, electrophoresed, transferred to nitrocellulose membrane, hybridised with the human c-*myc* gene; c-H-*ras* gene; β-actin gene. Lane *1*, NIH3T3 cells; Lane *2*, pP1-4 cells; Lane *3*, pT22-3 cells; Lane *4*, NIH3T3-nm-1 cells; Lane *5*, pT22-3-nm-2 cells.

**Figure 3** Expression of Glutathione S-Transferase-π gene and mdr-1 gene by Northern blot analysis. Lane *1*, NIH3T3 cells; Lane *2*, pP1-4 cells; Lane *3*, pT22-3 cells; Lane *4*, NIH3T3-nm-1 cells; Lane *5*, pT22-3-nm-2 cells; Lane *6*, PC-9/CDDP cells; Lane *7*, K562/ADM cells.

and K562/ADM as positive controls showed the expressions of the GST-π (at 0.8 kb) and mdr-1 (at 4.5 kb) genes, respectively. In contrast NIH3T3 cells and transfectants showed negligible expressions of both GST-π and mdr-1 genes.

*Drug sensitivity of transfectants*

Figures 4 and 5 show representative response curves for each cell line exposed to hCPA and melphalan. The cell lines transfected with the c-*myc* oncogene are more resistant than the parental NIH3T3 and N8 cells to these two agents. Each curve was obtained from the average of at least three independent experiments and the $IC_{50}$ (the concentration of drug which reduces the cell growth to 50% of control) for each cell line was calculated as the average of at least three independently-obtained $IC_{50}$s. Table II summarises the $IC_{50}$s of various drugs for all the cell lines. N8, transfected with pKOneo only, showed no significant difference in $IC_{50}$s compared with those of NIH3T3 cells to any of the anticancer agents. The cell lines transfected with the c-*myc* gene (NIH3T3-nm-1 and pT22-3-nm-2) were significantly more resistant than in the NIH3T3 and N8 cell line to cisplatin, melphalan, adriamycin, 4-hydroperoxycyclophosphamide, and CPT-11. With VP-16 there was no significant difference in $IC_{50}$s between any of the transfectants and NIH3T3 cells, but a trend towards resistance was obtained. The cell lines transfected with c-H-*ras* oncogene (pP1-4 and pT22-3) showed no significant difference in $IC_{50}$s compared with NIH3T3 and N8 cells to cisplatin and topoisomerase

**Figure 4** Sensitivity of NIH3T3 cells and transfectants to 4-hydroperoxycyclophosphamide. The symbols used in this figure are as follows: —△— NIH3T3; —▲— N8; —○— NIH3T3-nm-1; —●— pT22-3-nm-2.

**Figure 5** Sensitivity of NIH3T3 cells and transfections to melphalan. The symbols used are as follows: —△— NIH3T3; —▲— N8; —○— NIH3T3-nm-1; —●— pT22-3-nm-2.

**Table II** $IC_{50}$ values of anticancer drugs for transfectants

| | $IC_{50}$ values ($\mu g\ ml^{-1}$)[a] | | | | | |
|---|---|---|---|---|---|---|
| *Cells* | *CDDP* | *hCPA* | *Melphalan* | *ADR* | *VP-16* | *CPT-11* |
| NIH3T3 | $0.21 \pm 0.03$[b] | $0.34 \pm 0.02$ | $0.95 \pm 0.05$ | $0.21 \pm 0.03$ | $0.06 \pm 0.01$ | $9.64 \pm 0.26$ |
| N8[c] | $0.26 \pm 0.09$ | $0.59 \pm 0.15$ | $1.20 \pm 0.43$ | $0.22 \pm 0.06$ | $0.04 \pm 0.03$ | $9.09 \pm 0.82$ |
| nm-1[d] | $0.44 \pm 0.03$[f] | $1.60 \pm 0.18$[f] | $4.63 \pm 0.91$[g] | $0.34 \pm 0.03$[g] | $0.07 \pm 0.01$ | $16.2 \pm 3.25$[h] |
| nm-2[e] | $0.34 \pm 0.04$[g] | $1.11 \pm 0.37$[h] | $8.60 \pm 0.20$[f] | $0.46 \pm 0.06$[g] | $0.21 \pm 0.20$ | $21.2 \pm 0.73$[g] |
| pP1-4 | $0.26 \pm 0.02$ | $0.60 \pm 0.09$[g] | $15.40 \pm 1.54$[h] | $0.12 \pm 0.02$ | $0.06 \pm 0.01$ | $9.07 \pm 1.51$ |
| pT22-3 | $0.11 \pm 0.01$[g] | $0.71 \pm 0.03$[g] | $2.67 \pm 0.61$ | $0.07 \pm 0.01$ | $0.04 \pm 0.00$ | $7.07 \pm 0.67$ |

[a]$IC_{50}$ values are obtained as the concentrations which inhibit control cell growth by 50%; [b]Mean ± s.d. Values were calculated from the results obtained by at lest three independent experiments; [c]N8 cell line was obtained by the transfection of pKOneo plasmid alone to NIH3T3 cells; [d]NIH3T3-nm-1; [e]pT2203-nm-2; [f]$P < 0.001$; [g]$P < 0.01$; [h]$P < 0.05$.

inhibitors (adriamycin, VP-16 and CPT-11). With alkylating agents (hCPA and melphalan) the transfectants with c-H-*ras* had higher $IC_{50}$ values than those of the parental and N8 cells, although that for melphalan in pT22-3 was not significantly different. From these results it is concluded that (1) c-*myc* oncogenes increased the intrinsic resistance to the anticancer agents, cisplatin, 4-hydroperoxycyclophosphamide, adriamycin, melphalan and CPT-11 and (2) c-H-*ras* oncogenes increased resistance to alkylating agents only (3) G418 selection did not lead to the isolation of resistant clones which had activated various stress response genes.

### Discussion

The MTT assay has the advantage of short incubation time and simplicity. The assay can reflect both cytotoxic and growth inhibitory effect of anticancer agents, but, with longer cell doubling times, it mainly reflects cytotoxic effect. It is therefore important to determine the plated cell numbers and the incubation time in order to achieve an optimal evaluation of drug sensitivity. Following determination that each cell line could maintain linearity in its growth curve and yield accurate reproducibility of optical density, we decided to use a 4-day incubation period and different plating cell numbers, as described in Materials and methods.

All transfectants used in this study were obtained from the same parental NIH3T3 cells. Cells transfected with the c-*myc* oncogene acquired resistance to anticancer agents in that $IC_{50}$ values for all the drugs except VP-16 in nm-1 and nm-2 were significantly higher than those in parental NIH3T3 and N8 cells. Transfection of activated c-H-*ras* did not affect drug sensitivity in nm-2 considering that $IC_{50}$ values of pT22-3, transfected with activated c-H-*ras*, were not higher than those of NIH3T3 and N8 cells except for hCPA. In the two c-*myc* expressing lines (nm-1 and nm-2) there is a large difference in the amount of c-*myc* mRNA expressed, but no difference in the degree of drug resistance. One possible explanation for this observation is that the cotransfection of activated c-H-*ras* gene in nm-2 has some influence on drug sensitivity in this line. Another possibility is that there is a threshold in the amount of c-*myc* gene expression which influences drug sensitivity and that subclone variation does not always give rise to the difference in drug resistance among transfectants. We obtained another c-*myc* transfected cell line (nm-9) by the same procedure and this showed about the same $IC_{50}$ values to CDDP, hCPA, VP-16 and CPT-11 as those for nm-1. Moreover the $IC_{50}$ value of nm-9 for melphalan was 2.3 times higher than that of nm-1 and 9.8 times higher than that of the parental cells. These findings confirm that independently isolated c-*myc* subclones show the aquisition of resitance to CDDP, hCPA, melphalan and CPT-11 compared with the parental and pKOneo transfected cells. Further study is necessary to examine the correlation between drug resistance and the expression of c-*myc* oncogene by using not cotransfectants but a range of transfectants with different amounts of c-*myc* oncogene alone.

In order to determine how we can overcome resistance to anticancer agents, the genetic basis of such resistance is the most urgent issue that needs addressing (Anonymous, 1987; Busch, 1987). With regard to *myc* family genes, amplification of the N-*myc* oncogene is associated with rapid progression in neuroblastoma (Seeger *et al.*, 1985).

Additionally small cell lung cancer patients have a shorter survival period if their cells show c-*myc* gene amplification, and c-*myc* amplification is associated with a more virulent variant type of small cell lung cancer cell line (Johnson *et al.*, 1987*b*). Such cell lines transfected with c-*myc* oncogene show morphological changes in culture (Johnson *et al.*, 1986) and have shorter doubling times (Gazdar *et al.*, 1985). These observations therefore suggest that *myc* family genes are associated with some biological characteristics of malignant tumours, but do not directly prove that these genes contribute to the resistance to anticancer drugs. In the present study we have clearly demonstrated that transfection with the c-*myc* gene is associated with increased resistance to cisplatin, adriamycin, melphalan, CPT-11 and 4-hydroperoxycyclophosphamide.

This is the first report showing that c-*myc* oncogene can increase resistance to anticancer agents and there have been no reports to elucidate the mechanism of increase in drug resistance by c-*myc* oncogenes. It has been reported that transfection with the c-H-*ras* gene could increase the resistance to cisplatin and radiation in cell lines (Sklar, 1988*a*,*b*). On the other hand Toffoli *et al.* (1989) reported that transfection with H-*ras* did not induce resistance to CDDP, VP-16, Mitomycin C on adriamycin. In the present study we have demonstrated that the cell lines transfected with the c-H-*ras* gene increased resistance to some alkylating agents, but not to cisplatin, VP-16 and adriamycin. Although we have no data for Mitomycin C, our findings are not always inconsistent with the results of Toffoli. Previous findings have suggested that a mechanism of resistance to anticancer agents might be a decrease in intracellular accumulation of drugs. On the other hand, as in v-H-*ras* oncogene-transfected cells (Burt *et al.*, 1988), c-*myc* oncogenes might increase the expression of other genes known to be involved in drug resistance. There have been some reports that the overexpression of the GDT-π gene is associated with the acquisition of resistance to cisplatin (Nakagawa *et al.*, 1988) and adriamycin (Cowan, 1986). Our results in Table II show that nm-1 transfected with c-*myc* oncogene acquired a multidrug resistance phenotype. However, no apparent expression of GST-π and mdr-1 genes could be found in NIH3T3 cells or any of the transfectants. These results therefore indicate that resistance to anticancer agents in c-*myc* transfected cells cannot be explained by mdr-1 and GST-π gene expression. The c-*myc* transfected cell lines acquired higher resistance to alkylating agents than to other drugs (Table II, Figures 4 and 5). Considering that the target of alkylating agents is mainly DNA in the cell nucleus and that c-*myc* protein is primarily located in the nucleus, we are planning to study the accumulation of anticancer agents and examine DNA damage and repair in c-*myc* transfected cells as possible mechanisms of resistance.

This work was supported in part by a grant-in-aid from the Ministry of Health and Welfare for a Comprehensive 10-year Strategy of Cancer Control and by Grants-in-Aid for Cancer Research from the Ministry of Health and Welfare and from the Ministry of Education Science and Culture.

### References

ANONYMOUS (1987). Gene amplification in malignancy (editorial). *Lancet*, **i**, 839.

BELL, D.R., GERLACH, J.H., KATRER, N., BUICK, R.N. & LING, V. (1985). Detection of P-glycoprotein in ovarian cancer: a molecular marker associated with multidrug resistance. *J. Clin. Oncol.*, **3**, 311.

BRADLEY, G., NAIK, M. & LING, V. (1989). P-glycoprotein expression in multidrug-resistant human ovarian carcinoma cell lines. *Cancer Res.*, **49**, 2790.

BURT, R.K., GARFIELD, S., JOHNSON, K. & THORGEIRSSON, S.S. (1988). Transformation of rat liver epithelial cells with v-H-ras or v-raf causes expression of MDR-1, glutathione-S-transferase-π and increased resistance to cytotoxic chemicals. *Carcinogenesis*, **9**, 2329.

BUSCH, H. (1987). Oncogenes and other new targets for cancer chemotherapy. *National Library of Medicine*, **78**, 309.

CHOMCZYNSKI, P. & SACCHI, N. (1986). Single-step method of RNA isolation by acid guanidium thiocyanate-phenol-chloroform extraction. *Anal. Biochem.*, **162**, 156.

COWAN, K.H., BATIST, G., TULPULE, A., SHINHA, B.K. & MEYERS, C.E. (1986). Similar biochemical changes associated with multidrug resistance in human breast cancer cells and carcinogen-induced resistance to xenobiotics in rats. *Proc. Natl Acad. Sci. USA*, **83**, 9328.

DELORBE, W.J., LUCIW, P.A., GOODMAN, H.M., VARMUS, H.E. & BISHOP, J.M. (1980). Molecular cloning and characterization of avian sarcoma virus circular DNA molecules. *J. Virol.*, **36**, 50.

FASANO, O., BIRNBAUM, D., EDLUND, L., FOGH, J. & WIGLER, M. (1984). New human transforming genes detected by a tumorigenicity assay. *Mol. Cell Biol.*, **4**, 1695.

FUJIWARA, Y., SUGIMOTO, Y., KASAHARA, T. & 4 others. Determinant of drug response in a cisplatin resistant human lung cancer cell line. *Jpn. J. Cancer Res.* (in press).

GAZDAR, A.F., CARNEY, D.N., NAU, M.M. & MINNA, J.D. (1985). Characterization of variant subclasses of cell lines derived from small cell lung cancer having distinctive biochemical morphological and growth properties. *Cancer Res.*, **45**, 2924.

GOLDFARB, M., SHIMIZU, K., PERUCHO, M. & WIGLER, M. (1982). Isolation and preliminary characterization of a human transforming gene from T24 bladder carcinoma cells. *Nature*, **296**, 404.

HONG, W.S., SAIJO, N., SASAKI, Y. & 6 others (1988). Establishment and characterization of cisplatin resistant sublines of human lung cancer lines. *Int. J. Cancer*, **41**, 462.

JOHNSON, B.E., IHDE, D.C., MAKUCH, R.W. & 6 others (1987). Myc family oncogene amplification in tumor cell lines established from small cell lung cancer patients and its relationship to clinical status and course. *J. Clin. Invest.*, **79**, 1624.

JOHNSON, B.E., NAU, M., GAZDAR, A.F. & 4 others (1986*a*). Amplification of myc oncogenes is less common in small cell lung cancer patients tumors than in small cell lung cancer lines. *Proc. Am. Soc. Clin. Oncol. Annu. Meet.*, **4**, 16.

JOHNSON, B.E., BATTEY, J., LINNOLIA, I. & 5 others (1986*b*). Changes in the phenotype of human small cell lines after transfection and expression of the c-*myc* proto-oncogene. *J. Clin. Invest.*, **78**, 525.

KANO, T., SASAKI, M. & MURAMATSU, M. (1987). Structure and expression of a human class π Glutathione Transferase messenger RNA. *Cancer. Res.*, **47**, 5626.

LITTLE, C.D., NAU, M.M., CARNEY, D.N., GAZDAR, A.F. & MINNA, J.D. (1983). Amplification and expression of the c-myc oncogene in human lung cancer cell lines. *Nature*, **306**, 194.

MA, D.D., DAVEY, R.A., HARMAN, D.H. & 5 others (1987). Detection of multidrug resistant phenotype in acute non-lymphoblastic leukemia. *Lancet*, **i**, 135.

MANIATIS, T., GRITSCH, E.F. & SAMBROOKK, J. (1982). *Molecular Cloning: A Laboratory Manual.* Maniatis, T., Fritch, E.F. & Aambrook, J. (eds). Cold Spring Harbor Laboratory: New York.

MOSMANN, T. (1983). Rapid colorimetric assay for cellular growth and survival: application to proliferation and cytotoxicity assays. *J. Immunol. Methods*, **65**, 55.

NAKAGAWA, K., YOKOTA, J., WADA, M. & 4 others (1988). Levels of glutathione S-transferase π mRNA in human lung cancer cell lines correlative with the resistance to cisplatin and carboplatin. *Jpn. J. Cancer Res.*, **79**, 301.

NAU, M.M., BROOKS, B.J. & RATTERY, J. (1985). L-myc, a new myc related gene amplified and expressed in human small cell lung cancer. *Nature*, **318**, 69.

PERUCHO, M., GOLDFARB, M., SHIMIZU, K., LAMA, C., FOGH, J. & WIGLER, M. (1981). Human-tumor-derived cell lines contain common and different transforming genes. *Cell*, **27**, 467.

RONINSON, I.B., CHIN, J.E., CHOI, K. & 6 others (1986). Isolation of human mdr DNA sequences amplified in multidrug resistant KB carcinoma cells. *Proc. Natl Acad. Sci. USA*, **83**, 4538.

ROTHBERG, P.G., ERISMAN, M.D., DIEHL, R.E., ROVIGATTI, U.G. & ASSTRIN, M. (1984). Structure and expression of the oncogene c-myc in fresh tumor material from patients with hematopoietic malignancies. *Mol. Cell Biol.*, **4**, 1096.

SANTOS, E., TRONICK, S.R., AARONSON, S.A., PULCIANI, S. & BARBACID, M. (1982). T24 human bladder carcinoma oncogene is an activated form of the normal human homologue of BALB-and Harvey-MSV transforming genes. *Nature*, **298**, 343.

SCHIMKE, R.T. (1984). Gene amplification, drug resistance, and cancer. *Cancer Res.*, **44**, 1735.

SEEGER, R.C., BRODEUR, G.M. & 5 others (1985). Association of multiple copies of the N-myc oncogene with rapid progression of neuroblastoma. *N. Engl. J. Med.*, **313**, 114.

SKLAR, M.D. (1988*a*). Increased resistance to cis-Diamminedichloroplatinum(II) inm NIH3T3 cells transformed by ras oncogenes. *Cancer Res.*, **48**, 793.

SKLAR, M.D. (1988*b*). Ras oncogene increase the intrinsic resistance NIH3T3 cells to ionizing radiation. *Science*, **239**, 645.

SLAMON, D.J., CLARK, G.M., WENG, S.G., LEVIN, N.J., ULLRICH, A. & MCGUIRE, W.L. (1987). Human breast cancer, correlation of relapse and survival with amplification of the HER-2/neu oncogene. *Science*, **235**, 177.

SHIBUYA, M. & YAMAGUCHI, S. (1987). Structure and biological activity of the amplified c-myc gene in a transplantable human gastric cancer. *5th International Workshop of Immune-Deficient Animals, Copenhagen*. pp. 166–174, (1985) (Karger, Basel 1987).

SOUTHERN, E.H. (1975). Detection of specific sequences among DNA fragments separated by gel electrophoresis. *J. Mol. Biol.*, **98**, 503.

TOFFOLI, G., VIEL, A., TUMIOTTO, L., BUTTAZZI, P., BISCONTIN, G. & BOIOCCHI, M. (1989). Sensitivity pattern of normal and Ha-ras transformed NIH3T3 fibroblasts to antineoplastic drugs. *Tumori*, **75**, 423.

VIOLA, M.V., FROMOWITZ, F., ORAVEZ, S., DEB, S. & SCHLOM, J. (1985). Ras oncogene p21 expression is increased in premalignant lesions and high grade bladder carcinoma. *J. Exp. Med.*, **161**, 1213.